# Arginine vasopressin deficiency following SARS-CoV-2 infection: a rare pituitary complication

**DOI:** 10.1530/EDM-26-0006

**Published:** 2026-08-03

**Authors:** Pascoe Mannion, Edward Jude

**Affiliations:** ^1^ Department of Endocrinology, Tameside and Glossop Integrated Care, NHS Foundation Trust, Ashton-under-Lyne, UK; ^2^ Division of Cardiovascular Sciences, Faculty of Biology, Medicine and Health, The University of Manchester, Manchester, UK; ^3^ Division of Medicine and Manchester Academic Health Science Centre, Manchester University NHS Foundation Trust Manchester, Manchester, UK

**Keywords:** pituitary, rare diseases/syndromes

## Abstract

**Summary:**

We describe a rare case of arginine vasopressin deficiency (AVP-D) in a female in her 40s that developed two weeks following symptomatic COVID-19 infection. Symptoms included polydipsia, nocturia and polyuria of 9.1 L per day. A water deprivation test demonstrated an ongoing high urine output despite increasing plasma osmolality, confirming the diagnosis. Desmopressin was commenced with good effect and titrated to clinical response. This demonstrates the importance of awareness of potential pituitary complications following COVID-19 infection, including AVP-D.

**Learning points:**

- Arginine vasopressin deficiency can occur in the weeks following COVID-19 infection and should be suspected in patients with new-onset polyuria, polydipsia and nocturia.
- Key diagnostic markers include a raised plasma osmolality, low urine osmolality and high urine output.
- Desmopressin responsiveness should be assessed as part of a water deprivation test and dosage titrated to clinical response.
- A full pituitary profile should be performed in these patients to exclude additional pituitary pathology.

## Background

Coronavirus disease 2019 (COVID-19) is the disease caused by infection with the severe acute respiratory syndrome coronavirus 2 (SARS-CoV-2) virus. While infection manifests itself predominantly as an acute respiratory illness, many affected patients also develop persistent, long-term symptoms affecting a variety of organ systems following resolution of the acute phase, often termed ‘long COVID’. One area of particular interest to endocrinologists is the impact on the pituitary gland, with previous case studies describing hypopituitarism, pituitary apoplexy and syndrome of inappropriate antidiuretic hormone developing shortly following COVID-19 infection (1).

SARS-CoV-2’s entry into the cell is dependent on the binding of its spike protein to the ACE2 cell receptor (2). Notably, ACE2 has also been found to be expressed in the pituitary gland (3). However, at present, it is unclear whether COVID-19-associated pituitary dysfunction is mediated by direct viral invasion and destruction, or by an immune-mediated inflammatory response against the pituitary gland precipitated by acute infection.

One specific syndrome resulting from pituitary dysfunction is arginine vasopressin deficiency (AVP-D). AVP-D is defined by an insufficient production of arginine vasopressin from the posterior pituitary, with resulting loss of activation of the V2 receptor in the collecting ducts of the kidney causing significant polyuria and natriuresis. Herein, we describe a case of a patient presenting with AVP-D developing two weeks following COVID-19 infection.

## Case presentation

A female patient in her 40s was referred to our endocrinology service due to a 5-month history of polyuria, polydipsia, dry mouth and nocturia of approximately four times per night. The past medical history was significant for asthma and stress urinary incontinence. Regular medications included solifenacin, which had been commenced for the management of urinary frequency. No significant psychiatry history or previous lithium therapy was reported. Of note, the patient reported that these symptoms had commenced approximately two weeks after the development of symptomatic COVID-19 diagnosed via lateral flow test. Reported presentation of COVID-19 was mild, with symptoms including sore throat, cough, fever and dysgeusia. The patient reported that this was her second such episode of COVID-19 infection. No acute medical intervention or hospitalisation was required for either episode. A daily fluid chart was recorded as an outpatient, which charted a daily oral fluid intake of 9.8 L and urine output of 9.1 L. Given this patient’s excessive oral fluid intake and urine output, and a raised plasma osmolality on previous laboratory investigations, a diagnosis of AVP-D was suspected.

## Investigation

Initial blood tests were performed (see Table 1) demonstrating a normal plasma sodium of 142 mmol/L, an adjusted calcium of 2.44 mmol/L (9.8 mg/dL) and an eGFR of 89.2 mL/min/1.73 m^2^. However, other biochemical tests taken showed an elevated plasma osmolality of 300 mmol/kg, a low urine osmolality of 113 mmol/kg and a urine sodium of 25 mmol/L. A glucose and HbA1c were also performed, which were 4.8 mmol/L (86 mg/dL) and 36 mmol/mol (5.4%), respectively, thus ruling out diabetes mellitus. Thyroid function tests were euthyroid, with a TSH of 3.34 mIU/L and free T4 of 10.4 pmol/L (0.81 ng/dL). Plasma cortisol was also sent with a borderline result of 259 nmol/L (9.4 µg/dL), but subsequent ACTH stimulation testing demonstrated an increment to 561 nmol/L (20.3 µg/dL). Other pituitary profile bloods included plasma prolactin (151 mIU/L), IGF-1 (70 nmol/L (535 ng/mL)), FSH (5.5 IU/L) and LH (3.7 IU/L), which were all unremarkable.

**Table 1:** Baseline laboratory investigations performed at initial presentation.

Investigation	Results	Normal range
Haematology		
Haemoglobin (g/L)	137	120–150
White blood count (×10^9^/L)	6.7	4.0–10.0
Platelets (×10^9^/L)	295	150–410
Biochemistry		
Plasma sodium (mmol/L)	142	133–146
Plasma potassium (mmol/L)	4.5	3.5–5.3
Plasma urea (mmol/L)	3.6	2.5–7.8
Plasma creatinine (μmol/L)	71	41–86
Estimated GFR (mL/min/1.73 m^2^)	89.2	>90
Plasma-adjusted calcium (mmol/L)	2.44	2.12–2.63
Plasma glucose (mmol/L)	4.8	3.0–7.8
HbA1c (mmol/mol)	36	<41
Plasma osmolality (mmol/kg)	300	275–295
Urine osmolality (mmol/kg)	113	250–750
Urine sodium (mmol/L)	25	40–220
Endocrinology		
Plasma TSH (mIU/L)	3.34	0.4–4.5
Plasma free T4 (pmol/L)	10.4	7.0–17.0
Pre-Synacthen plasma cortisol (nmol/L)	253	172–497
Post-Synacthen plasma cortisol (nmol/L)	561	>420
Plasma prolactin (mIU/L)	151	102–496
Plasma IGF-1 (nmol/L)	70	59–210
Plasma FSH (IU/L)	5.5	
Follicular phase		3–13
Mid-cycle		9–18
Luteal phase		1–10
Plasma LH (IU/L)	3.7	
Follicular phase		2–15
Mid-cycle		22–90
Luteal phase		1–19

GFR, glomerular filtration rate.

As such, this patient proceeded to undergo an inpatient water deprivation test (see Table 2A). Baseline measurements included a blood pressure of 115/58 mmHg, weight of 78.4 kg, urine output of 470 mL/h, plasma osmolality of 291 mmol/kg and urine osmolality and sodium of 120 mmol/kg and 18 mmol/L, respectively. Following 6 h of fluid deprivation, there was a slight increase in the patient’s urine osmolality to 332 mmol/kg, which had remained submaximal despite plasma osmolality reaching 304 mmol/kg at maximum. Weight had decreased to 76.2 kg, blood pressure to 112/56 mmHg, and urine output to 135 mL/h. A dose of 2 μg of intravenous desmopressin was then given, which achieved an increase in urine osmolality to 604 mmol/kg and urine sodium to 96 mmol/L, a reduction in the urine output to 30 mL/hour and normalisation of the plasma osmolality to 295 mmol/kg (see Table 2B). A diagnosis of AVP-D was made, and the patient started on desmopressin therapy.

**Table 2:** (A) Water deprivation test. Baseline observations and investigations were taken, and then, hourly measurement of weight, urine volume, blood pressure, plasma osmolality and urine osmolality and sodium was performed. Unfortunately, due to an error in clinical documentation, only 6 h of water deprivation testing results were available. (B) Desmopressin responsiveness test. Following completion of water deprivation test, 2 μg of intravenous desmopressin were administered. Hourly measurement of weight, urine volume, blood pressure, plasma osmolality and urine osmolality and sodium was then performed as previously stated.

Tests	0 min	60 min	120 min	180 min	240 min	300 min	360 min
A. Water deprivation test							
Weight (kg)	78.4	78.0	77.2	76.8	76.6	76.5	76.2
Urine volume (mL)	470	490	300	255	125	125	135
Blood pressure (mmHg)	115/58	103/55	105/57	102/56	105/53	106/57	112/56
Plasma osmolality (mmol/kg)	291	301	304	303	299	298	297
Urine osmolality (mmol/kg)	120	71	102	145	267	316	332
Urine sodium (mmol/L)	18	12	21	35	59	73	77
B. Desmopressin responsiveness test							
Weight (kg)		76.7	77.5	77.7	78		
Urine volume (mL)		40	30	-	20		
Blood pressure (mmHg)		110/58	107/73	117/56	110/53		
Plasma osmolality (mmol/kg)		300	294	291	295		
Urine osmolality (mmol/kg)		460	542	571	604		
Urine sodium (mmol/L)		61	62	69	96		

**Figure 1:**
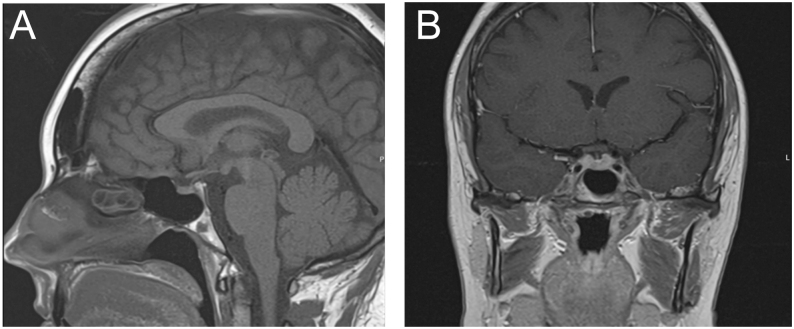
T1-weighted MRI of the pituitary demonstrating normal pituitary morphology in both sagittal (A) and coronal (B) views.

Following the water deprivation test, an MRI of the pituitary was also performed, which was unremarkable (see Fig. 1), with a normal pituitary morphology measuring 13.5 × 9.5 × 5.7 mm in transverse, anteroposterior and craniocaudal lengths. No focal lesions or thickening of the pituitary stalk was noted. A repeat MRI of the pituitary performed 11 months later also showed a normal appearance of the pituitary, with no evolving lesions since the previous scan. Given the lack of space-occupying or infiltrative lesions on imaging, a presumptive diagnosis was made of a COVID-19-related autoimmune or post-infectious process as the underlying cause of her AVP-D.

## Treatment

Initial therapy was started with 100 μg of oral desmopressin twice daily with a significant impact on thirst, fluid intake and urinary output. However, there was noted to be development of thirst with high fluid intake and urinary output in the evening in-between doses of desmopressin. This was suspected to be due to a low trough plasma concentration of desmopressin prior to the evening dose, and the desmopressin dosing was increased to 100 μg three times per day, with good effect on these residual symptoms.

## Outcome and follow-up

After 12 months of follow-up, this patient has continued to achieve good symptom control on her current dose of desmopressin, with a normal daily urine output of approximately 1–1.5 L per day, a normal serum sodium of 138 mmol/L and no further significant nocturia. She is currently under investigation for mild bifrontal headaches that are felt to be unrelated to her AVP-D.

## Discussion

We describe the case of a patient with mild symptomatic COVID-19 infection who developed new onset of AVP-D in the post-infectious period. The onset of AVP-D in this case was reported to be around two weeks following the onset of infection – this is consistent with existing cases of AVP-D in the literature, which report a range in time to onset of 12 days to 3 months (4, 5, 6, 7, 8, 9). Similarly to this patient, most of these cases also occurred following resolution of the acute phase. However, three cases described in the literature developed AVP-D while hospitalised during the acute phase of their illness, one of which was already intubated and ventilated at development of AVP-D due to acute respiratory distress syndrome (4, 5, 6).

While this patient presented with AVP-D in isolation, other cases of COVID-19-related AVP-D have been noted to have additional post-infectious phenomena that were felt to also be related to COVID-19 infection, including myocarditis and central adrenal insufficiency (6, 7). Interestingly, with the exception of the case of central adrenal insufficiency (6), no other cases have been identified describing concomitant onset of other pituitary pathologies alongside AVP-D. MRI of the pituitary in this patient was unremarkable. However, two of the other described cases were noted to have an absent posterior pituitary bright spot associated with thickening of the pituitary stalk on the imaging suggestive of infundibuloneurohypophysitis, with one of these cases also having anti-rabphilin-3A antibodies identified on western blot (8, 9). It is unclear, however, whether all these described cases of post-COVID-19 AVP-D share a common autoimmune aetiology, or other pathological processes, such as direct viral invasion and destruction of arginine vasopressin-expressing hypothalamic neurons.

In this case, the timing of onset between COVID-19 infection and AVP-D, the lack of other identified causes of AVP-D or concomitant pituitary dysfunction and the presence of other similar cases within the literature lead us to conclude that a COVID-19-related pathological process was responsible for the development of AVP-D. This patient presented with a typical clinical picture of AVP-D with polyuria, polydipsia and nocturia alongside a raised plasma osmolality and reduced urine osmolality. While this patient did have some reduction in urine output and an increase in urine osmolality during the water deprivation test, this was likely due to the direct effects of dehydration resulting in reduced fluid delivery to the glomerulus. The reduction in weight, increase in plasma osmolality, submaximal urine osmolality and excellent response to desmopressin during this test all point strongly towards an underlying diagnosis of AVP-D.

Patients with COVID-19 infection of any severity presenting with onset of polyuria and polydipsia should have a potential diagnosis of AVP-D considered. Hyperglycaemia and concomitant diabetes mellitus should also be investigated for and excluded in these patients, especially given the use of dexamethasone as a treatment for severe COVID-19 infection. While most cases are idiopathic, it is recommended to exclude other underlying causes and dysfunction of other pituitary hormones. Desmopressin is an appropriate treatment in those where responsiveness is demonstrated and should be titrated based on clinical response.

## Declaration of interest

The authors declare that there is no conflict of interest that could be perceived as prejudicing the impartiality of the study reported.

## Funding

This research did not receive any specific grant from any funding agency in the public, commercial or not-for-profit sector.

## Patient consent

Written consent for publication of their clinical details was obtained from the patient.

## Author contribution statement

Both authors were involved in providing clinical care for the patient. PM performed the literature review, collected the data and wrote the majority of the manuscript, including figures and tables. EJ performed the initial endocrinological evaluation of the patient and provided revision and editing of the completed manuscript.
